# Epilepsy Mechanisms in Neurocutaneous Disorders: Tuberous Sclerosis Complex, Neurofibromatosis Type 1, and Sturge–Weber Syndrome

**DOI:** 10.3389/fneur.2017.00087

**Published:** 2017-03-17

**Authors:** Carl E. Stafstrom, Verena Staedtke, Anne M. Comi

**Affiliations:** ^1^Division of Pediatric Neurology, Department of Neurology, Johns Hopkins University School of Medicine, Baltimore, MD, USA; ^2^Department of Neurology, Kennedy Krieger Institute, Johns Hopkins University School of Medicine, Baltimore, MD, USA

**Keywords:** seizure, epilepsy, neurocutaneous disorder, tuberous sclerosis complex, neurofibromatosis, Sturge–Weber syndrome, mechanistic target of rapamycin

## Abstract

Neurocutaneous disorders are multisystem diseases affecting skin, brain, and other organs. Epilepsy is very common in the neurocutaneous disorders, affecting up to 90% of patients with tuberous sclerosis complex (TSC) and Sturge–Weber syndrome (SWS), for example. The mechanisms underlying the increased predisposition to brain hyperexcitability differ between disorders, yet some molecular pathways overlap. For instance, the mechanistic target of rapamycin (mTOR) signaling cascade plays a central role in seizures and epileptogenesis in numerous acquired and genetic disorders, including several neurocutaneous disorders. Potential routes for target-specific treatments are emerging as the genetic and molecular pathways involved in neurocutaneous disorders become increasingly understood. This review explores the clinical features and mechanisms of epilepsy in three common neurocutaneous disorders—TSC, neurofibromatosis type 1, and SWS.

## What is a Neurocutaneous Disorder?

Neurocutaneous disorders are a heterogeneous group of genetic disorders characterized by abnormalities of the cutaneous and nervous systems. Tuberous sclerosis complex (TSC), neurofibromatosis type I (NF1), and Sturge–Weber syndrome (SWS) are prototypical neurocutaneous disorders in which genetic mutations in pathways regulating cell growth cause developmental dysfunction of the brain, skin, and other organs. Clinically, these neurocutaneous disorders differ significantly, but certain similarities also exist. Namely, all neurocutaneous disorders are congenital, affect several organs, are associated with learning or developmental problems, persist lifelong, are currently uncorrectable, and are optimally managed with a multidisciplinary approach in which neurologists, oncologists, educational specialists, neuropsychologists, and other therapists work together to monitor for potential complications of the respective disease and maximize abilities. In addition, there is now emerging evidence of some overlap in the cellular signaling pathways in these disorders (1–3). Epilepsy, defined as the condition of recurrent, unprovoked seizures, is a common feature of many of the neurocutaneous disorders. This chapter reviews aspects of the clinical presentation and management of epilepsy in TSC, NF1, and SWS and focuses on possible mechanisms of seizures and epilepsy in each disorder. Emphasis is placed on why epilepsy is so prevalent in these conditions.

## Mechanisms of Seizures and Epilepsy

A seizure is an episode of transient neurological dysfunction due to abnormal firing of neurons. Epilepsy is the condition of recurrent, unprovoked seizures. An epilepsy syndrome involves a specific seizure type(s) plus other features such as age of onset, electroencephalographic (EEG) findings, genetics/natural history, and responsiveness to particular drugs. A seizure occurs when there is an imbalance between excitation (E) and inhibition (I) in one or more areas of the brain. An E/I imbalance can manifest at the level of subcellular signaling pathways, ion channels, synapses, or neuronal networks. Any molecular, cellular, or structural pathology that increases excitation or decreases inhibition can generate a seizure (4). For example, enhanced activity or function of excitatory glutamatergic synapses or their receptor subtypes [i.e., alpha-amino-3-hydroxy-5-methyl-4-isoxazolepropionic acid (AMPA) or *N*-methyl-d-aspartate (NMDA)] or decreased activity or function of inhibitory gamma-aminobutyric acid (GABA)-ergic neurons or receptors would favor seizure generation. Similarly, dysfunction of sodium or calcium channels (often excessive current flow through these channels) or diminished function of potassium channels may predispose to hyperexcitability and seizures.

Epilepsy or the process by which epilepsy develops (epileptogenesis) also entails E/I imbalance but other cellular pathologies can also be involved, such as structural or circuit rearrangements, inflammation, or disrupted epigenetic regulation. Therefore, the mechanisms underlying a seizure and those leading to epilepsy overlap but epileptogenesis also involves additional processes. Though admittedly oversimplified, the concept of an imbalance between excitation and inhibition is helpful when considering seizure and epilepsy mechanisms in neurocutaneous disorders, and pathophysiological changes can be considered at each of these levels of neuronal function (4, 5).

## Tuberous Sclerosis Complex

### Clinical Features of TSC

Tuberous sclerosis complex is a multiorgan system disorder occurring at a frequency of about 1 in every 5,000 births. The disorder is caused by a mutation in *TSC1* or *TSC2*, which are tumor repressor genes controlling the activity of the mechanistic target of rapamycin (mTOR) signaling pathway (formerly called mammalian target of rapamycin), discussed further below. Overactivity of the mTOR pathway accelerates mRNA translation and causes excessive protein synthesis and cell growth, leading to the formation of benign tumors in several organs and giving rise to the distinctive clinical features of TSC. For this reason, TSC is considered the prototypical “mTOR-opathy.” Full diagnostic criteria for TSC have been published recently (6, 7). Classic skin findings include hypopigmented macules (ash-leaf spots) and facial angiomatoses. In addition, patients with TSC commonly have cardiac rhabdomyomas (often present at birth with subsequent involution) and renal angiomyolipomas (develop over time and frequently lead to renal insufficiency in childhood or adulthood). Here, we focus on central nervous system abnormalities in TSC that may lead to seizures.

**Figure 1:**
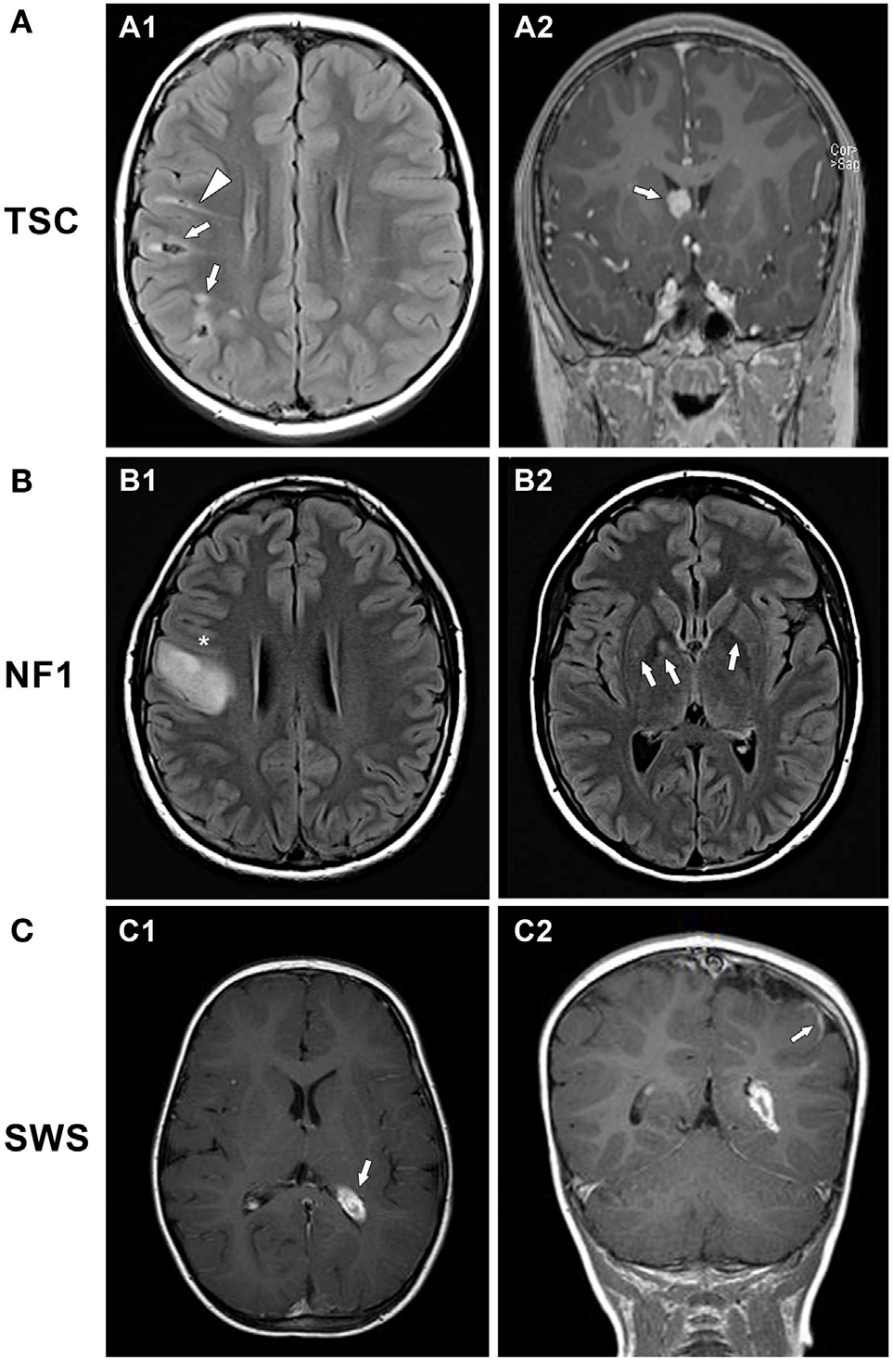
**Examples of brain MRI scans in patients with neurocutaneous disorders**. **(A)** 8-year-old boy with tuberous sclerosis complex. **(A1)** Axial fluid attenuation inversion recovery (FLAIR) and **(A2)** coronal contrast-enhanced T1-weighted images show multiple cortical/subcortical, partially calcified mixed FLAIR-hyper/hypointense tubers in the right front parietal lobes (arrows, A1), FLAIR-hyperintense migration lines (triangle, A1), and a homogeneously enhancing, round subependymal giant cell astrocytoma within the right foramen of Monro (arrow, A2). **(B)** 16-year-old girl with neurofibromatosis type 1. **(B1)** Axial FLAIR and T1 post-contrast images showing a large expansile lesion centered at the right precentral gyrus (*) indicating a low-grade glioma. **(B2)** Axial T2 FLAIR image reveals focal areas of signal intensity (arrows) in the right globus pallidus and putamen and similar but to a lesser extent on the contralateral side, representing nonspecific unidentified bright objects. **(C)** 21-month-old boy with Sturge–Weber syndrome. **(C1)** Axial T1-weighted post-contrast image showing left hemisphere atrophy with prominent choroid plexus glomus (arrow). **(C2)** Coronal T1-weighted post-contrast image with occipital-parietal leptomeningeal enhancement (arrow).

Three major neuropathological findings characterize TSC (Figure 1A1). First, hamartomas (tubers) of widely variable size and number form in the cerebral cortex, often at the gray–white matter junction. Tubers are composed of a mixture of abnormal cells, including dysplastic, immature, cytomegalic neurons and glia, all lacking a normal lamination pattern. Importantly, tubers do not tend to expand or grow. Although tubers are considered to be static lesions, some dynamic features have been documented and tubers may appear to become more prominent over time due to interval myelination and other factors (8, 9). Tubers are extremely epileptogenic and can produce one or more seizure foci in the brain. A seizure can arise from the tuber itself or from perituberal cortex adjacent to the tuber (10, 11). Tubers differ in their epileptogenicity, even within the same patient. The precise localization of the epileptogenic focus is critical for planning tuber resection surgery. The number, volume, and possibly location of tubers correlate to some extent with the patient’s level of intellectual impairment and cognitive dysfunction (12, 13). For example, tubers located in the temporal lobe are associated with a high incidence of autism (14, 15). Second, abnormal neuronal and glial tissue, called subependymal nodules (SENs), can arise in the periventricular regions. SENs can transform into subependymal giant cell astrocytomas (SEGAs). Due to their location near the foramen of Monro, SEGAs can cause acute blockage of cerebrospinal fluid flow at that site, leading to hydrocephalus (Figure 1A2). However, owing to their cellular composition and location deep in the brain, SEGAs are not epileptogenic. Third, radially oriented heterotopias occur within white matter, consistent with disordered neuronal migration; these heterotopias have been associated with behavioral problems and may also contribute to epileptogenesis (Figure 1A1) (16). Together, these three neuropathological features account for the major age-dependent neurologic complications of TSC, namely, epilepsy (90%), autism (25–50%), and intellectual disability (40–80%). The common occurrence of neuropsychological deficits in TSC, including learning disability, attention deficits, spatial memory problems, aggressive behaviors, anxiety, and sleep disruption, has given rise to the term TAND (tuberous sclerosis-associated neuropsychiatric disorders) (17). Optimal management of patients with TSC must address TAND as well as epilepsy.

### Epilepsy in TSC

Seizures are extremely common in TSC, affecting up to 90% of patients (18). In 63% of TSC patients with epilepsy, the seizures appear in the first year of life; 80% of seizures begin before 3 years of age and 70% eventually become refractory to drugs. The seizures may be focal, multifocal, infantile spasms, or a combination of these or other seizure types. Infantile spasms are a seizure type, a subtype of epileptic spasms occurring in the first year of life, and often occur as part of the electroclinical syndrome, West syndrome. Focal seizures may be isolated or occur in association with infantile spasms, and most cases of infantile spasms also include other seizure types. The incidence of infantile spasms is extremely high, affecting approximately one-third of children with TSC (18, 19). In about 5% of children with TSC, spasms continue or develop after 2 years of age, making this a very common semiology (20). With multiple tubers comprising potential seizure foci, it is not surprising that focal or multifocal seizures are present in TSC, but the frequent occurrence of infantile spasms has not been explained adequately. It is difficult to categorize infantile spasms solely as generalized or focal seizures, as both occur, even in TSC. Thus in the case of TSC, the common final output—spasms—can obviously be produced by focal or multifocal lesions. Abnormal mTOR function may be another explanation for infantile spasms, but mTOR dysfunction is seen in only a subset of the numerous etiologies of infantile spasms, so mTOR pathway dysfunction is not required for infantile spasms to occur.

Earlier seizure onset portends a less favorable outcome with regard to neuropsychiatric and developmental function (17). In TSC, the interaction between epilepsy and neuropsychiatric sequelae is complex, compounded by adverse effects of the therapies used to treat epilepsy. As opposed to classic epileptic encephalopathies in which seizures themselves cause cognitive impairment beyond the etiology, in TSC it is the molecular etiology—mTOR overactivation—that probably drives both the epilepsy and the encephalopathy/TAND (21).

The treatment of seizures in TSC involves conventional antiseizure drugs as well as non-pharmacological interventions such as the ketogenic diet, vagus nerve stimulation, or in some cases, surgical resection of the epileptogenic tubers (13, 22, 23). The percentage of patients with drug-refractory epilepsy in TSC (~70%) is much higher than the percentage of drug-refractoriness among patients with epilepsy in general (~30%) (24). Early surgical resection is recommended if a dominant tuber is identified, i.e., an area from which the majority of seizures appear to originate, but the presence of multiple tubers often makes it difficult to localize the most epileptogenic one. This identification may be aided by the use of positron emission tomography (PET) scans, which can localize brain regions with abnormal metabolism even in the absence of a structural lesion (25). The goal of surgery is palliation of seizure burden, though cognition can also improve after the targeted resection of an epileptogenic tuber (26). A meta-analysis of 229 patients in 13 studies concluded that 59% of patients were seizure free after surgery (27).

For infantile spasms in children with TSC, vigabatrin is the first choice medication, rather than adrenocorticotropic hormone or corticosteroids as used more commonly for infantile spasms due to other etiologies. The vigabatrin response rate is between 73% and 96% (28). It is unknown why vigabatrin works so well in TSC-associated infantile spasms. The therapeutic benefit could be related to vigabatrin’s main mechanism of action—inhibition of GABA transaminase, leading to increased availability of the inhibitory neurotransmitter GABA at the synapse—or to vigabatrin-induced decrease in mTOR activation (29). [However, the situation may not be so simple, as mTOR is upregulated by the excessive GABA present when the gene for succinic semialdehyde dehydrogenase is knocked out in mice (mimicking human SSADH deficiency) (30).] Since infants with TSC have such a high risk for developing infantile spasms, a novel approach is to treat infants who have epileptiform abnormalities on EEG (but no documented seizures) prophylactically with vigabatrin before spasms begin (31, 32). This approach is being tested in an ongoing European multicenter trial called EPISTOP.

Mechanistic target of rapamycin inhibition could comprise a rational, disease-modifying treatment for seizures in TSC (2, 9, 13, 23). mTOR inhibitors such as rapamycin (sirolimus) have been found to decrease the size of kidney angiomyolipomas and SEGAs (33) and are being trialed for seizures as well (23). Another mTOR inhibitor, everolimus, has shown benefit for seizures in TSC in a phase I/II clinical trial (34). In that trial, seizure frequency decreased by at least 50% in 12 of 20 participants and the median decrease in seizures was 73% in 17 of the 20 patients; in addition, there was a decrease in seizure duration and improved quality of life on several parent-reported measures. A recent phase III double-blind randomized study of 366 TSC patients with treatment-resistant focal-onset seizures (EXIST-3) compared placebo *versus* low- or high-dose everolimus (35). The median reduction in seizure frequency was 40% in those receiving high-dose everolimus and 28% in subjects receiving low-dose everolimus, both statistically significant compared with a 15% seizure reduction in the placebo group. Notably, children with infantile spasms were excluded from these studies. These results raise the possibility that mTOR inhibition decreases seizure frequency in TSC by targeting the molecular defect, representing a unique, disease-modifying approach. This and other therapies are urgently needed for epilepsy in TSC, emphasizing the importance of understanding the mechanisms of epileptogenesis in this disorder (36).

### Molecular Basis of TSC—Overactivation of the mTOR Pathway

Tuberous sclerosis complex arises *de novo* in approximately 70–80% of cases; in the remainder of cases, inheritance is autosomal dominant or due to genetic mosaicism. The disorder is caused by a mutation in one of the tumor suppressor genes, *TSC1* or *TSC2*. *TSC1*, located on chromosome 9, encodes the protein hamartin. *TSC2*, located on chromosome 16, encodes the protein tuberin. Under normal circumstances, hamartin and tuberin act together as a dimer to inhibit the mTOR signaling pathway and constrain cell growth and differentiation (37). mTOR is a serine/threonine kinase that acts as a central regulator of cell growth, differentiation, proliferation, and migration. Mutation of either *TSC1* or *TSC2* leads to the clinical features of TSC, which are variably expressed due to incomplete penetrance of the mutated gene. It is thought that tuber/hamartoma development requires “two hits,” whereby a germline mutation in one allele of *TSC1* or *TSC2* is complemented by a second somatic mutation in the other allele, leading to cell growth derangement and hamartoma formation (38). Overall, *TSC2* mutations confer worse seizures and cognition than *TSC1* mutations (20, 39, 40).

**Figure 2:**
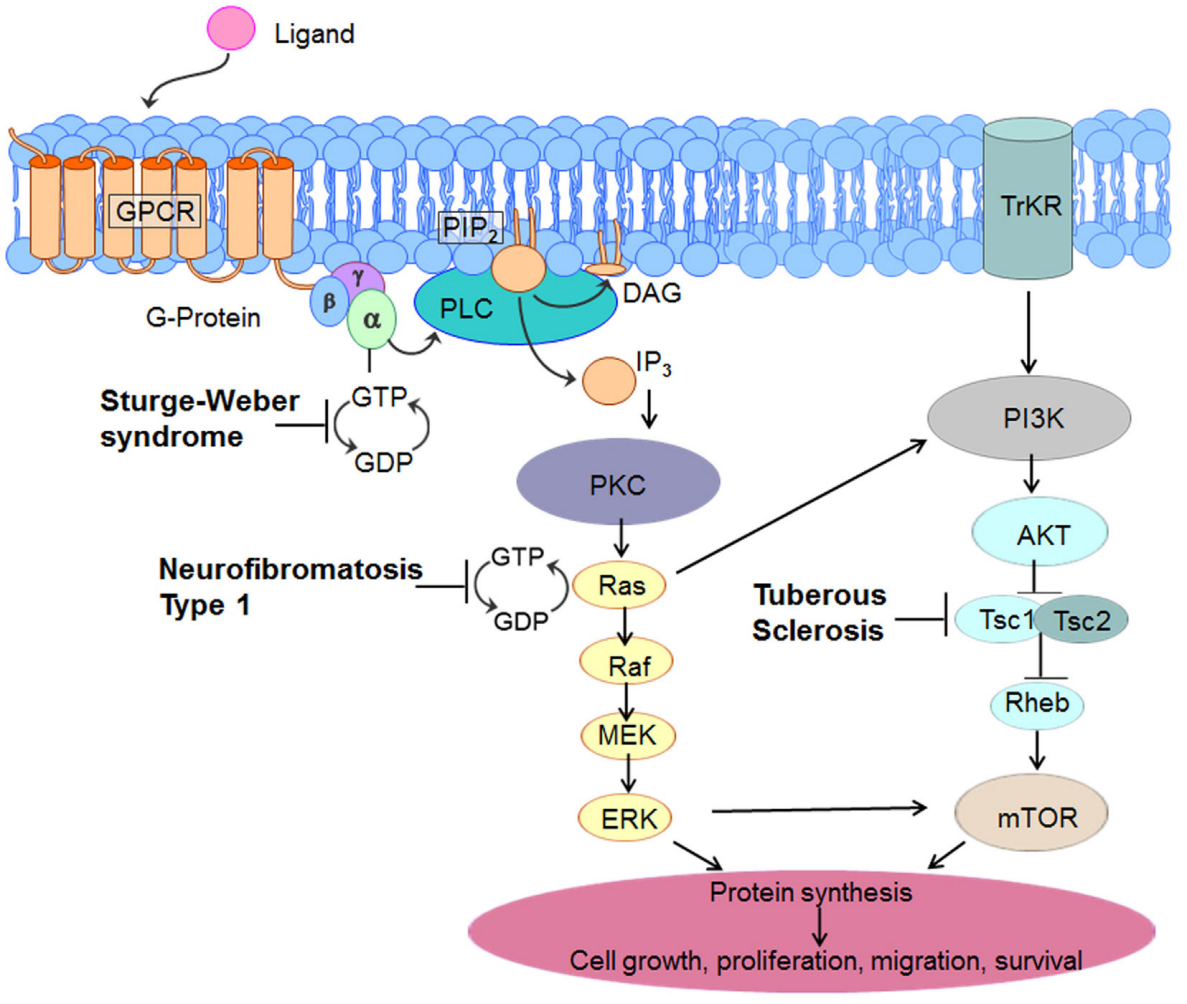
**Molecular pathways involved in three common neurocutaneous disorders— tuberous sclerosis complex (TSC), neurofibromatosis type 1, and Sturge**–**Weber syndrome**. As elaborated in the text, this diagram indicates unique sites of dysfunction as well as interactive and overlapping pathways that might serve as target-specific interventions.

The mTOR signaling pathway begins at the cell membrane, where receptors respond to growth factors and nutrient/energy molecules (Figure 2). Phosphatidylinositol triphosphate 3-kinase (PI3K) activates protein kinase B (AKT) which is a serine/threonine-specific protein kinase and potent pro-survival and pro-oncogenic protein. AKT directly phosphorylates *TSC2* and inhibits its function. *TSC2* inactivation by AKT reduces Ras homolog enriched in brain (Rheb), a small GTPase that is a member of the Ras (*ra*t *s*arcoma) superfamily. Overexpression of Rheb due to a *TSC1* or *TSC2* mutation leads to increased mTOR activation and excessive cell growth and proliferation. These effects are blocked by rapamycin, which inhibits the mTOR pathway by binding directly to mTOR (mTOR complex 1 or mTORC1), thereby decreasing phosphorylation of downstream mTOR effectors (41, 42). These downstream effectors include two key regulators of protein translation, ribosomal S6-kinase (S6K) and eukaryote initiation factor 4E binding protein 1 (4E-BP1). The loss of *TSC1* or *TSC2* function leads to selective hyperactivation of the mTOR cascade, resulting in mTOR-dependent increased phosphorylation of S6K and 4E-BP1 proteins (43). Therefore, these gene mutations provide a plausible mechanism to account for the characteristic giant cells (cytomegaly) in TSC. Inhibition of this cascade by mTOR inhibitors results in growth suppression and restricted cell size.

Aside from its control of cell growth and survival, mTOR is also involved in synaptic plasticity, learning and memory (44). Long-term potentiation (LTP), a process whereby repeated activation of a neural pathway strengthens the connections between neurons, is considered to be a cellular correlate of learning and memory. mTOR activation is involved in the late phase form of LTP that is dependent on protein synthesis. The impairment of LTP by seizures is a candidate mechanism for cognitive impairment in epilepsy (45, 46). In *TSC1* conditional knockout mice, *TSC1^+/−^*, the LTP deficit is partially reversed by NMDA receptor antagonists, and it has been suggested that LTP impairment is due to overactivation of the mTOR pathway or NMDA receptors (i.e., by seizures) (47, 48). Likewise, in *TSC2^+/−^* mice, rapamycin improves synaptic plasticity (LTP) and reduces behavioral deficits (49).

In addition to its pivotal role in the pathogenesis of TSC, mTOR dysfunction also plays a role in other neurologic disorders, both genetic (hemimegalencephaly, focal cortical dysplasia) and acquired (temporal lobe epilepsy, traumatic brain injury) (37, 48, 50–52). mTOR’s central action on cell growth regulation occurs in other neurocutaneous disorders as well, as discussed below for NF1 and SWS.

### Epilepsy Mechanisms in TSC

**Figure 3:**
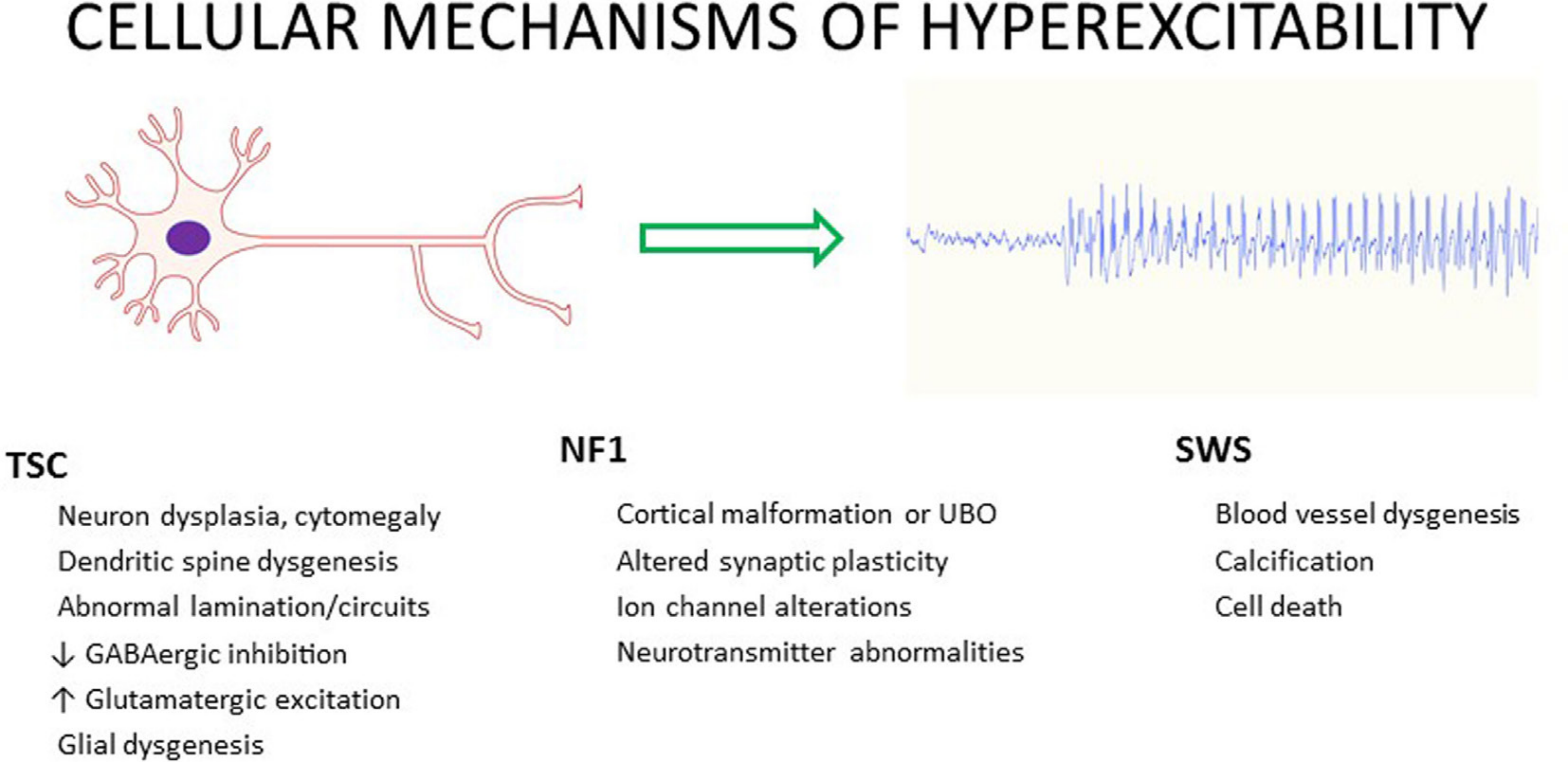
**Possible contributors to cellular hyperexcitability and seizures in three common neurocutaneous disorders—tuberous sclerosis complex (TSC), neurofibromatosis type 1 (NF1), and Sturge**–**Weber syndrome (SWS)**.

In TSC, the mechanisms of E/I imbalance leading to seizure generation and epileptogenesis are complex and multifactorial, related to both the dysfunction of the mTOR signaling pathway (abnormal cellular excitation) and the neuropathological substrates (hyperexcitable circuits) (53, 54). TSC pathophysiology may be summarized as follows: *abnormal molecules in abnormal cells form abnormal circuits, together leading to increased seizure propensity and epileptogenesis* (55). This summary statement emphasizes the involvement of pathophysiological factors at multiple levels of brain function. In TSC, numerous features could contribute to the propensity for epilepsy by altering E/I balance, including altered cerebral cortical architecture, astrocyte proliferation, calcification, altered vascular anatomy, edema, altered neurotransmitter receptor expression, and cell proliferation and death (Figure 3) (42).

Favoring excess excitation, glutamate receptors in dysplastic neurons and giant cells have increased expression of NMDA receptor subunits NR2B and NR2D (in newer nomenclature, GluN2B and GluN2D) and the neuronal glutamate transporter EAAC1 (56). In *TSC1^+/−^* mice, there is persistent, mTOR-dependent selective functional upregulation of the NMDA receptor subunit GluN2C, specifically in cortical layer IV neurons that are critical for thalamocortical integration of excitatory activity; this receptor subtype mediates excessive current through NMDA channels and enhances recurrent excitation and seizure propensity; furthermore, seizure generation is blocked by GluN2C antagonists (57). Tubers resected from children with TSC have a selective increase in the expression of AMPA receptor subunits GluR1 and GluR2 in dendrites of dysplastic neurons compared with normal neurons, suggesting that increased calcium permeability engendered by these receptor subunit alterations could promote epileptogenesis (58). Mice lacking *TSC1* also exhibit abnormally long excitatory postsynaptic currents and epileptic discharges, like human tubers (59).

Favoring decreased inhibition, tubers have lower levels of the GABA synthetic enzyme glutamate decarboxylase 65, the vesicular GABA transporter VGAT, and GABA_A_ receptor subunits α1 and α2 (56). In cortical specimens from patients with TSC, the number of GABAergic interneurons is decreased, consistent with diminished and aberrant local inhibition (60). Excessive depolarizing GABA responses in tuberal neurons were blocked by bumetanide, a compound that inhibits the ion transporter sodium-potassium-chloride cotransporter 1 (NKCC1) (61, 62). Microtransplantation of cortical membranes from TSC patients into *Xenopus* oocytes revealed a lack of expected maturation of both GABA_A_ receptor function (delayed transition to hyperpolarizing action) and a persistently elevated ratio of NKCC1 to the potassium-chloride cotransporter 2 (KCC2, whose expression is more prominent beyond the neonatal stage and is responsible for the hyperpolarizing action of GABA receptor activation); also, AMPA receptor subunit expression (GluR1/GluR2 ratio) was increased compared to non-TSC control tissue that did undergo developmental maturation, suggesting a pattern of cerebral dysmaturity in TSC (63). Ion channel dysfunction might also cause hyperexcitability in TSC—in astrocytes of *TSC1*-deficient mice, there is decreased expression of potassium inward rectifier channels (Kir2.1 and Kir 6.1), limiting the passage of hyperpolarizing current (64). Collectively, these observations suggest that different tuber components possess different molecular profiles that could alter the E/I balance in favor of excitation and predispose to seizures.

In addition to molecular alterations, mTOR overactivity, *via TSC1* and *TSC2* mutation, leads to structural aberrations in neurons and dendrites and their growth. The soma size and dendrite size and density of hippocampal pyramidal cells are perturbed by the loss of a single copy of *TSC1* (65). Additional mechanisms that may play a role in seizure predisposition in TSC include somatic and dendritic hypertrophy, aberrant basal dendritic structure, and enlargement of axon tracts. These changes lead to increased synaptogenesis and recurrent circuit formation (66). E/I imbalance may be promoted by mTOR-dependent repression of inhibitory input onto excitatory hippocampal pyramidal neurons, resulting in excitatory positive feedback (67). Attesting to the role of the mTOR pathway and its inhibition in disorders other than TSC, in a rodent model of temporal lobe epilepsy, rapamycin reduces the sprouting of mossy fiber axons that in part underlies hyperexcitability in the hippocampal dentate gyrus and may underlie epilepsy progression (68). Furthermore, rapamycin directly augments inhibition by increasing potassium channel (Kv1.1) expression (69) and decreases excitation by reducing the surface expression of AMPA receptors (70). Rapamycin also reduces the duration of epileptiform bursts induced by the GABA_A_ receptor antagonist, bicuculline (71).

Animal models of TSC, created by deletion or knockout of *TSC1* or *TSC2* in certain cell types, have provided considerable insight into mechanisms by which seizures and epilepsy occur in TSC, as indicated by several experimental observations already discussed. Mice with conditional knockout of glial fibrillary acidic protein (GFAP), a marker of glial cells, have increased mTOR activity and increased SK6 leading to enhanced epileptogenicity. Importantly, brains of these knockout animals have no tubers (72). Therefore, epilepsy in these mice occurs without requiring the presence of a tuber or other macroscopic structural lesion, suggesting that alteration of the mTOR pathway itself is sufficient to generate seizures. The function of the glutamate transporters GLT and GLAST is decreased in astrocytes of these mice (73). The result is altered glutamate homeostasis with excessive extracellular and synaptic glutamate levels, leading to excitotoxicity *via* increased excitatory postsynaptic potentials and increased NMDA receptor activation, which increase seizure propensity. Acute seizures activate the mTOR pathway transiently (74), whereas status epilepticus leads to chronic mTOR elevation (75). There is also evidence of increased inflammatory markers such as IL-1β in GFAP knockout mice, and treatment with anti-inflammatory drugs leads to decreased seizures and increased survival (76). Timing of mTOR inhibition may be important—early treatment with rapamycin decreases mTOR activation, decreases glial proliferation, increases GLT1 expression, prevents epilepsy, and increases survival. Late rapamycin treatment, given after the mice have already developed spontaneous seizures, is also beneficial in decreasing seizure frequency and increasing survival (72). It remains a mystery why patients with TSC have such a high predilection to infantile spasms. The lack of animal models of infantile spasms hinders exploration of the underlying pathophysiology (77). In the “multiple-hit” model of infantile spasms, high-dose rapamycin delivered in daily pulses for 3 days ameliorates spasm occurrence (78); this is not a TSC model *per se*, rather one that mimics symptomatic spasms as a consequence of brain injury induced by three toxins. These results implicate the mTOR pathway as a possible treatment target in symptomatic infantile spasms and emphasize the possible effectiveness of pulse mTOR inhibition as opposed to continuous rapamycin administration.

## Neurofibromatosis Type 1

### Clinical Features of NF1

Neurofibromatosis type 1, the most common neurocutaneous disorder, occurs in approximately 1 in 3,000 births (79). Inheritance is autosomal dominant and the mutations are *de novo* in about half the cases. The diagnosis of NF1 is clinical, based on consensus criteria (80), and confirmation with genetic testing is available but usually not necessary (81). Disease manifestations are quite diverse, even within a family, related to variable penetrance of the mutation. Characteristic findings are hyperpigmented skin markings or café-au-lait macules (which typically develop in the first 2 years of life), axillary freckling, disease-specific Lisch nodules (hamartomas of the iris), optic pathway gliomas, and neurocognitive deficits. NF1 patients are also at risk to develop multiple benign and malignant Schwann cell neoplasms such as tumors of the peripheral nerve sheath, gliomas, pheochromocytoma, gastrointestinal stromal tumors, and other cancers including breast cancer in young women and leukemia (82). Neurofibromas, the hallmark of the disease, are benign nerve sheath tumors of two types, dermal (which can be cutaneous or subcutaneous) or plexiform. Both types may grow and cause pain and disfigurement; however, only plexiform neurofibromas can undergo malignant transformation into a malignant peripheral nerve sheath tumor, an aggressive spindle-cell sarcoma with poor prognosis (83). Optic pathway gliomas are very common, affecting up to 15% of children with NF1, the majority presenting before 7 years of age. Gliomas of the hemispheres, brainstem, or cerebellum can also occur, which are mostly pilocytic or diffuse astrocytomas (Figure 1B1). In addition to gliomas, central nervous system lesions in NF1 frequently include T2-hyperintensities on MRI scan, known as “unidentified bright objects” (UBOs) (Figure 1B2) (84). Autopsy studies have demonstrated that UBOs are vacuolar changes in myelin sheaths with dysplastic glial proliferation, often seen in the cerebellum, basal ganglia, subcortical white matter, and thalamus (85). The etiopathogenesis of UBOs is unknown; their presence may be associated with poorer cognition, but the data remains conflicted (86, 87). Intriguingly, UBOs often disappear by adulthood and their role in nervous system dysfunction is unclear (88). Also, cortical malformations, in the form of disordered cortical architecture, have been documented (85, 89).

Cognitive deficits in NF1 tend to be mild or moderate and include learning problems in more than half the patients, and these can be significant enough to affect academic performance and quality of life. Although no specific learning disability is characteristic of NF1, common deficits affect visual-spatial and visual motor abilities, executive function, verbal and nonverbal language, fine and gross motor coordination, and attention (90–92). Sleep disorders and anxiety are very common. Migraine headaches occur in up to 33%, autism in 15%, and epilepsy in 6–10% (93). Megalencephaly, manifesting as macrocephaly, is due to increased white matter volume (94). Other organ systems often involved in NF1 include skeletal (long bone dysplasia, scoliosis) and vascular (blood vessel stenosis, especially the renal artery; aneurysms; hypertension).

### Epilepsy in NF1

While epilepsy is much less common in NF1 than in TSC, its incidence of 6–10% is still considerably greater than that of epilepsy in the general population (0.5%). Seizures in NF1 tend to have focal onset and commonly generalize secondarily (95–97). It is thought that seizures in NF1 arise from the numerous focal lesions that comprise the disorder, namely, tumors and malformations of cortical development (Figure 1B1). Thus, seizure occurrence requires neuroimaging, even if previous neuroimaging was normal. The relationship of UBOs to seizures is controversial, but most studies have concluded that UBOs are not associated with seizures (84, 96). Seizures in NF1 are often relatively easy to control with one or more conventional antiseizure drugs; surgical resection of offending lesions is sometimes pursued (98). Surgery has been most successful for temporal lobe gliomas (93).

### Molecular Basis of NF1—Ras Overactivation

The *NF1* gene on chromosome 17 encodes neurofibromin, a large cytoplasmic tumor suppressing protein. Neurofibromin, a GTPase-activating protein that suppresses the proto-oncogene Ras, is highly expressed in neurons and glia, especially oligodendrocytes. Neurofibromin ordinarily inhibits Ras activity by catalyzing the hydrolysis of GTP-bound Ras to GDP-bound Ras (converts the proto-oncogene p21-Ras from an active form to an inactive form), thereby preventing tumor formation (Figure 2) (37, 99). In NF1, the lack of neurofibromin leads to unopposed Ras (GTPase) activity, causing released control of downstream signals involved in cell growth and differentiation, such as mitogen-activated protein kinase (MAPK, also known as extracellular-signal regulated kinase or ERK) and mTOR. Activation of Ras leads to enhanced activation of Ras-PI3K, MAPK, and mTOR. Therefore, NF1 is considered a “Ras-opathy.” Neurofibromin controls mTOR *via* a common biochemical pathway with tuberin (100). In that sense, the molecular pathways in TSC and NF1 share some common features. mTOR overactivation has been demonstrated in NF1-associated tumors (99). However, the degree of mTOR activation in NF1 is modest compared to TSC, and it is not known how significant a role mTOR dysfunction plays in NF1 or NF1-associated seizures (3). Neurofibromin also positively regulates cyclic adenosine monophosphate (cAMP), and increased cAMP levels are associated with decreased cell growth (92).

In NF1, cognitive deficits appear to be related to synaptic dysfunction as a consequence of signaling dysfunction of Ras-ERK, cAMP, and dopamine homeostasis rather than to a macroscopic structural lesion. In mice with targeted disruption of *NF1* (heterozygous null mutations, *NF1^+/−^*), Schwann cells have increased Ras activation and increased growth rate. These mice have cognitive impairments that parallel many human NF deficits in visuospatial learning, working memory, attention, and motor skills (101). Despite a lack of structural brain abnormalities, these mutant mice demonstrate several deficits in cognitive function. *NF1^+/−^* mice have impaired LTP, constituting a cellular basis for their impaired ability to perform hippocampus-based tasks involving spatial learning and memory (102). Furthermore, the cognitive deficits in NF1 are thought to arise from increased GABAergic signaling and can be reversed in animal models by pharmacologically inhibiting Ras-ERK activity with statin drugs (e.g., lovastatin, simvastatin) (103, 104). Studies in Drosophila homozygous for null *NF1* gene mutations revealed a linkage between cAMP generation and neurofibromin as the underlying cause for neurobehavioral manifestations in NF1 (105), later confirmed in mammalian species (106). In these studies, decreased cAMP generation due to reduced levels of functional neurofibromin resulted in olfactory learning deficits. However, the role of cAMP in human cognitive dysfunction in NF1 patients remains less clear and further investigations are required. Lastly, reduced dopamine levels in the striatum have been described in *NF1^+/−^* mice and correction of the dopamine defect resulted in improved cognition whereas Ras inhibition was ineffective (107). Due to those multiple mechanisms underlying neurobehavioral deficits, it is not surprising that clinical trials of lovastatin in patients have had variable success (108). Some benefits in working memory and verbal memory were found in a recent 14-week randomized trial of lovastatin versus placebo (109).

### Epilepsy Mechanisms in NF1

Neurofibromin plays important roles in multiple aspects of cortical development, including synaptic plasticity, learning and memory, neurotransmitter phenotype, and synapse formation (92). However, it is unclear why brains of individuals with NF1 are hyperexcitable and predisposed to seizures, and this topic is rarely discussed in the literature (110, 111). Possibilities are admittedly speculative and comprise the spectrum of pathophysiologies that disrupt the E/I balance. Of possible relevance to seizure mechanisms, it was found that GABA release and levels are *enhanced* in *NF1^+/−^* mice, a result of loss of neurofibromin leading to unrestrained ERK signaling and enhanced synaptic GABA release (104). While this finding was used to explain the impaired cognition, learning, and LTP of *NF1^+/−^* mice, *decreased* rather than increased GABA levels would be more consistent with a predisposition to epilepsy. However, augmented GABA release strategically limited to local inhibitory circuits could theoretically enhance excitability.

Ion channels are also receiving increasing attention in NF1, both in the peripheral nervous system, where enhanced pain perception is a common clinical problem, and in the central nervous system, related to abnormal LTP and cognition (112). Calcium channel opening is enhanced, and calcium currents are increased in hippocampal neurons in *NF1^+/−^* mice (113), which would increase excitability and neurotransmitter release. Dysfunction of a variety of ion channels (e.g., sodium, potassium, hyperpolarization-activated cyclic nucleotide-gated) has been reported in different brain regions and NF models (112, 114), but no consistent pattern has emerged to put forward a unified hypothesis about cortical hyperexcitability or seizures. In peripheral nociceptors, several sodium channel isoforms (NaV1.1, NaV1.7, NaV1.8) have increased expression and activity in *NF1^+/−^* mice, leading to hyperexcitability (115). These findings may have relevance to central neurons and circuits, a topic ripe for investigation in terms of epilepsy mechanisms in NF. There is no published information as to whether *NF1^+/−^* mice have altered susceptibility to seizures induced by standard experimental methods (e.g., bicuculline, kindling).

## Sturge–Weber Syndrome

### Clinical Features of SWS

Sturge–Weber syndrome, with an estimated incidence of 1 in 20,000 live births, is a sporadically occurring neurocutaneous disorder characterized by vascular malformations of the brain, skin, and eyes (116). The clinical hallmarks are facial angiomas (port-wine birthmarks), malformations of leptomeningeal blood vessels, and ocular angiomas causing glaucoma. Children born with a facial port-wine birth mark in the region of the forehead, temple, and upper eyelid have a greatly increased risk of also having brain or eye involvement. The cerebral vascular malformations are typically unilateral (on the same side as the port-wine birthmark) but can also occur bilaterally, which confers a worse prognosis (117). Clinical manifestations include recurrent strokes and stroke-like episodes that can lead to hemiparesis, visual field defects, cognitive deterioration, and developmental delays involving language and behavior (116). Most infants develop fairly normally for some period of time prior to the onset of seizures. Children may manifest with early handedness or a gaze preference. Seizures most commonly present in the first year of life (118). Along with the capillary-venous leptomeningeal malformations, cortical malformations such as focal cortical dysplasia or polymicrogyria can also be seen (119, 120). The cerebral atrophy, calcification, and clinical deficits can be progressive, particularly in infants and young children. Later problems can include specific learning disabilities, attention deficit disorder, and hormone deficiencies (e.g., growth hormone, thyroid hormone) (121, 122).

### Epilepsy in SWS

Epilepsy occurs in 72% of SWS cases with unilateral cerebral involvement and 90% or more of those with bilateral involvement (117, 123). Seizures usually have a focal onset with secondary generalization. Although seizures often begin in the first year of life and generally by 2 years of age, about 10% can begin later in childhood, adolescence, or adulthood. The seizures commonly occur in clusters or as status epilepticus, with relatively long periods between bouts (124). The prolonged seizures in SWS are thought to worsen cognitive function (125). The combination of seizures very early in life (before 6 months of age), and extensive brain pathology portends a poor outcome. Seizures in SWS can be progressive as brain atrophy advances, leading to refractory epilepsy. Seizures sometimes respond to anticonvulsants such carbamazepine or oxcarbazepine; levetiracetam, also used commonly in SWS, can be effective at times but is associated with more frequent side effects (126). Topiramate and valproic acid are useful third-line agents. While topiramate should always be used with caution owing to the possibility that it can exacerbate glaucoma, children with SWS do not appear to be at increased risk for topiramate-associated glaucoma. In SWS, topiramate-associated glaucoma is an adverse effect seen more commonly, but not exclusively, in adults (127). Metabolic approaches such as the ketogenic or modified Atkins diet can be effective (128). Surgical options for refractory epilepsy include focal resection or hemispherectomy (129). Aspirin has been advocated to reduce stroke occurrence (130). mTOR inhibitors have not been studied in SWS. The high incidence of seizures in SWS raises the prospect that prophylactic treatment prior to the onset of seizures might improve developmental outcome, and clinical trials of this idea are needed (131).

### Molecular Basis of SWS—Abnormal Blood Vessel Formation

Sturge–Weber syndrome is caused by a post-zygotic somatic mosaic mutation of *GNAQ*, a gene that is critical for blood vessel development (132). Interestingly, *GNAQ* mutations are found in individuals who have the full spectrum of SWS with skin, eye, and brain involvement as well as in patients with isolated port-wine birth marks and no brain or eye involvement. Mutations earlier in development are presumed to be associated with more severe manifestations; an animal model needs to be developed to test this hypothesis. *GNAQ* codes for Gαq, the alpha subunit of a heterotrimeric G protein that activates downstream pathways including the ERK and mTOR pathways (Figure 2). Most recent evidence suggests that endothelial cells in the capillary malformations are particularly enriched in *GNAQ* mutations (133) but the precise mechanisms by which the mutation in endothelial cells results in the vascular malformations of SWS are not known. Expression of angiogenesis factors, such as vascular endothelial growth factor, hypoxia-inducible factor α1 (134), and fibronectin (135) is altered, and these factors likely play an important role in the pathophysiology. For example, *Elfn1* (extracellular-leucine-rich repeat fibronectin domain 1) is a gene enriched in hippocampal GABAergic interneurons that helps to recruit metabotropic glutamate receptors (e.g., mGluR7) to the presynaptic membrane; mutant mice with *Elfn1* knocked out develop seizures (136, 137).

### Epilepsy Mechanisms in SWS

The mechanism of epileptogenesis in SWS is uncertain, but abnormal blood vessel development undoubtedly figures prominently in the neuropathology and epilepsy pathogenesis (138). The clinical consequences of SWS are in part related to abnormal draining veins leading to venous stasis and congestion, which decreases regional perfusion and eventually causes hypoxic brain injury with neuronal loss and gliosis. Cortical malformations such as polymicrogyria with inherent circuit dysfunction also contribute to epilepsy pathogenesis (119). Seizures appear to originate in the cortex adjacent to the leptomeningeal angioma (139). Ictal single positron emitted computed tomography studies have demonstrated that prolonged seizures exacerbate perfusion deficits and may increase the risk of stroke (140). Diseased brain tissue shows decreased blood flow interictally and increased or decreased blood flow during a seizure. Positron emission tomography (PET) scans, which measure glucose uptake and therefore correlate with neuronal activity, have demonstrated interictal hypermetabolism in children with SWS, possibly indicating areas of incipient cortical dysfunction; these observations have implications for surgical planning (141). Fluorodeoxyglucose-PET and clinical studies show that if seizures are controlled for a prolonged period, glucose metabolism and neurodevelopmental status can improve (142). Increased vascular permeability from the leptomeningeal malformations likely leads to calcium leakage across the blood–brain barrier. While calcium itself is probably not epileptogenic, it certainly represents a marker for areas of cortical dysfunction.

An animal model would facilitate understanding of epilepsy pathogenesis in SWS, and efforts to create such a model are underway (143). However, there are several challenges to generating a model of a disorder with somatic mosaic mutations involving gain-of-function. The mutant gene must be expressed in the relevant cells and body structures only, a gene knockout approach is not applicable, and the developmental timing of the post-zygotic mutation is unknown. Nevertheless, molecular approaches to model creation using cell culture, mice, and other systems such as zebrafish should afford insights into seizure mechanisms as a consequence of endothelial cell maldevelopment (143). Continuing the theme already discussed for TSC and NF1, alterations in specific signaling molecules and pathways may engender hyperexcitable circuits in SWS, with mTOR dysfunction occupying a central location in the molecular pathways controlling numerous cell differentiation and migration (144). However, a role of mTOR in epileptogenesis in SWS has not been defined.

## Conclusion

Clinical and mechanistic aspects of epilepsy in TSC, NF1, and SWS are summarized in Table 1.

**Table 1:** **Epilepsy in common neurocutaneous disorders**.

Disorder	Gene mutation	Abnormal protein product	Epilepsy frequency	Most common seizure types	Molecular/cellular mechanism of epilepsy	Mechanism-specific therapy?
Tuberous sclerosis complex (TSC)	*TSC1* or *TSC2*	Hamartin or tuberin	70–90%	– Focal onset, may generalize – Infantile spasms	– mTOR overactivation – Cortical dysgenesis and tubers – K channels – GABA	– Vigabatrin – mTOR inhibitors
Neurofibromatosis (NF) type 1	*NF1*	Neurofibromin	6–10%	– Focal onset, may generalize	– Ras overactivation – Cortical malformations and dysgenesis – Neurofibromin role in cortical development	– Ras inhibitors
Sturge–Weber syndrome (SWS)	*GNAQ*	Gαq (GTPase)	70–90%	– Focal onset, may generalize, especially in clusters – Status epilepticus	– Abnormal blood vessel development – Cerebral calcifications – Neuronal loss – Astrogliosis – Cortical dysgenesis	– ASDs – ASA

*TSC, tuberous sclerosis complex; NF, neurofibromatosis; *GNAQ*, gene for guanine nucleotide-binding protein (G protein), subunit alpha, q polypeptide; Gαq, guanine nucleotide-binding protein (G protein), subunit alpha, q polypeptide; GTP, guanosine-5′-triphosphate; GABA, gamma-aminobutyric acid; mTOR, mechanistic target of rapamycin; Ras, rat sarcoma; ASD, antiseizure drug; ASA, acetyl salicylic acid (aspirin)*.

Targeted therapies are emerging from increased molecular understanding of neurocutaneous disorders, based on both unique molecular pathways for each syndrome and commonalities among syndromes [i.e., mTOR dysfunction (2, 3, 110)]. Clues to the pathophysiology of epilepsy in TSC, NF1, and SWS may arise from the observation that in each disorder, seizures have a focal onset, focusing attention on mechanisms altering the E/I balance at discrete sites of seizure initiation and subsequent spread. Figure 3 lists some of the factors that may lead to neuronal hyperexcitability in these neurocutaneous disorders.

Other insights might be gleaned from the very rare cases in which more than one neurocutaneous syndrome exists in a single individual. For example, a child was reported with maternal inheritance of NF1 and paternal inheritance of TSC (145). This girl developed intractable epilepsy at age 5 years and felbamate was the only antiseizure drug that helped. Since felbamate’s main mechanism of action is NMDA receptor antagonism and since NMDA downregulates the mTOR pathway, it was suggested that felbamate was acting specifically to dampen the mTOR pathway and reduce neuronal excitability. Such speculations are intriguing but need to be considered cautiously. A few patients with combined SWS and TSC have been reported, but none since the discovery of the somatic mutation causing SWS (146); it would be informative to better understand these dual mutations. There is no doubt, however, that epilepsy mechanisms in neurocutaneous disorders represent a vastly understudied topic that warrants concerted clinical and laboratory investigations. Indeed, epilepsy mechanisms may reveal new insights into the relationship between cognition, behavior, seizures, and other aspects of brain function in the neurocutaneous disorders (147).

## Author Contributions

CS, VS, and AC each contributed to the conceptualization, design, writing, and final approval of the manuscript, and each is accountable for all aspects of the work. AC drew Figure 2.

## Conflict of Interest Statement

The authors report no conflicts of interest or financial relationships relevant to this work.
